# Improving the Response Rate of Teaching Feedback by Introducing Exam Practice Questions – a Quality Improvement Project

**DOI:** 10.1192/bjo.2022.157

**Published:** 2022-06-20

**Authors:** Bruce Tamilson, Abigail Swerdlow, Sonya Rudra

**Affiliations:** 1 East London Foundation NHS Trust, London, United Kingdom; 2 Queen Mary University of London, London, United Kingdom; 3 Tavistock and Portman NHS Foundation Trust, London, United Kingdom

## Abstract

**Aims:**

Aim: The quality improvement (QI) project aimed to improve the response rate of teaching feedback from medical students at Queen Mary University of London (QMUL). *Background information: Universities and health care settings use students' feedback to improve the teaching and other services. The feedback is a valuable source to evaluate a service delivery and improvement. Following the COVID-19 pandemic a large majority of teaching switched to being held online. Feedback plays an important role in evaluating these new methods of teaching. However, response rates were noted to be low. This QI project aims to improve the response rate from students.*

**Methods:**

The project was registered on LifeQI and carried out during the psychiatric teaching for 4th year medical students at QMUL. The team emphasized the importance of feedback to students and produced online feedback forms which are mobile-friendly and concise. These were provided to students immediately after lectures and in an email reminder. As a change idea, five multiple choice practice questions from the topics of the day were included as a follow-on activity from the feedback form, with the expectation that this would motivate the students to complete the feedback. The response rate was calculated as a percentage (number of responses/number of attendees x 100%) and compared before and after the change was introduced using the independent t-test.

**Results:**

Introducing practice MCQs at the end of the feedback form resulted in a significant improvement: the response rate increased from 22.3% to 50%, more than doubled. The independent t-test found a significant increase in the number of feedback forms returned from the original rates (M = 13.8, SE = 3.0) to rates after practice questions were introduced into feedback (M = 30.6, SE = 1.7), t= -4.9 p = 0.001.

**Conclusion:**

Students’ motivation to complete feedback plays a major role in the response rate of medical students’ feedback at QMUL. Adding five MCQs on the topics of the day to the feedback form has significantly increased the response rate of 4th year medical students at QMUL. This project was limited to 4th year medical students who received online psychiatric lectures. It is important to try other change ideas in future in order to compare the outcomes.

